# Rethinking treatment paradigms for the deployment of SARS-CoV-2 antiviral drugs on the shifting landscape of new variants

**DOI:** 10.3389/fmicb.2022.998287

**Published:** 2022-10-12

**Authors:** Maxime Hentzien, Andrew Owen, Nathalie Strub-Wourgaft, Carmen Pérez-Casas, Marius Trøseid, Alexandra Calmy

**Affiliations:** ^1^HIV/AIDS Unit, Infectious Diseases Division, Geneva University Hospitals, Geneva, Switzerland; ^2^Centre of Excellence in Long-acting Therapeutics (CELT), University of Liverpool, Liverpool, United Kingdom; ^3^COVID Response and Pandemic Preparedness Director, Drugs for Neglected Diseases Initiative (DNDi), Geneva, Switzerland; ^4^Strategy, Unitaid, Geneva, Switzerland; ^5^Section of Clinical Immunology and Infectious Diseases, Oslo University Hospital, Oslo, Norway

**Keywords:** COVID-19, direct-acting antivirals, immunocompromised, resistance, variant emergence, monoclonal antibodies, omicron, variants

## Introduction

Monoclonal antibodies targeting the anti-SARS-CoV-2 spike (S) protein are prescribed in high-income countries to prevent severe disease in at-risk patients. Although studies report efficacy as between 50–85% (Weinreich et al., 2021; Gupta et al., 2022; Montgomery et al., 2022), global access is currently largely inequitable (Wiltz et al., 2022). Multivariant omicron (B.1.1.529) and subvariant (BA.2 followed by BA.4 and BA.5) dominance has challenged the treatment landscape for mild-to-moderate disease, introducing considerable uncertainty on the efficacy of monoclonal antibodies (Cao et al., 2022; Yamasoba et al., 2022) and leading to changes to initial recommendations for some of them (United States Food Drug Administration, 2022). Contemporaneously, oral, direct-acting antivirals with a reported efficacy ranging from 30% (molnupiravir) (Jayk Bernal et al., 2022) to 89–90% (nirmatrelvir/ritonavir) (United States Food Drug Administration, 2021) have recently received conditional or emergency approval in some countries and been recommended in international guidelines such as the World Health Organization guidelines (World Health Organization, 2022). S-217622, also known as ensitrelvir, a 3CL protease inhibitor that has been shown to significantly reduce the infectious viral load (Mukae et al., 2022a,b), is currently in phase 3 trials and waiting for emergency approval in Japan (Otake, 2022) and should be submitted soon in China (Notice Regarding the Initiation of the Submission of Preparation Materials for a New Drug Application for S-217622, a Therapeutic Drug for COVID-19, in China, 2022). The main purpose of this opinion paper is to highlight the possible strategies to optimize and protect current and future therapeutic options to treat the most vulnerable patients.

## Protecting emerging treatment options

**Figure 1:**
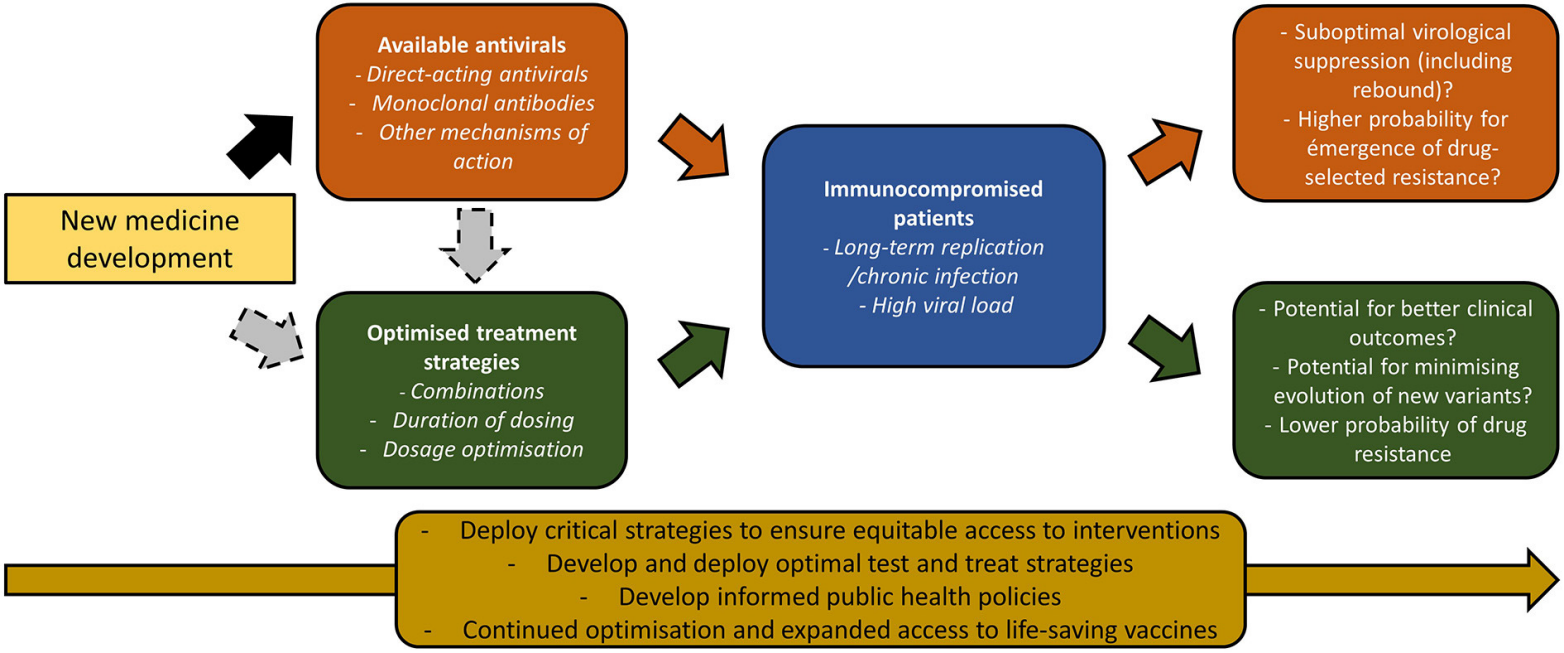
Potential impact of SARS-CoV-2 antiviral drugs optimization in protecting available antivirals in the shifting landscape of new variants.

Several crucial issues warrant urgent attention to optimize the use of these emerging treatment options (Figure 1). First, as proven to be transformational for HIV, rapid, affordable access to early antiviral treatment to slow the tide of new variants is critical to effective “test-and-treat” strategies to protect the most fragile patients and avoid a severe and/or persistent infection. After more than 2 years of pandemic, progress has been slow (Hasan et al., 2022) and public health attention has recently been attracted by the low-profile agreement during the (World Trade Organization, 2022) in Geneva in May 2022 (Financial Times, 2022). Together with vaccination, early diagnosis and treatment have the ability to reduce disease worsening, to reduce transmission and to constrain variability in viral sequences (United Kingdom Scientific Advisory Group for Emergencies, 2021).

Second, although the combined effect of omicron and increasing vaccine deployment in some regions has shifted the demand response from hospital to outpatient care, considerable uncertainty exists about who is now at risk for severe omicron disease (Skarbinski et al., 2022). While the risk/benefit ratio across at-risk subpopulations has unquestionably changed in vaccinated populations, gains made can only be preserved if those at highest risk are rapidly diagnosed and receive treatment in less than one week.

Third, high levels of antiviral efficacy will be critically important, especially in immunocompromised patients who are grossly underrepresented in registrational trials (John and John, 2020; Trøseid et al., 2022). Causes of immunosuppression are diverse (including organ/stem cell transplants, cancer, immunosuppressive medications or uncontrolled HIV) and these patients represent a significant proportion of the population, e.g., 7 million adults in the USA (Harpaz et al., 2016), but also in low- and middle-income countries due to the high prevalence of uncontrolled HIV. Overall, the mortality risk with omicron is still unclear, but protection of those who cannot be effectively vaccinated or protected by a prior SARS-CoV-2 infection remains imperative (Overvad et al., 2022). Importantly, in regions where HIV is highly prevalent, there is a clear need and opportunity to reinforce HIV epidemic control by prompt diagnosis and sustained viral suppression with antiretrovirals, key factors to also enable the control of SARS COV-2 spread in this group (Msomi et al., 2021; Meiring et al., 2022).

Although there are many other causes for variant emergence (host jump or adaptation, vaccine exposure, to name the most frequent), data confirm that immunocompromised patients with long-term SARS-CoV-2 replication are particularly susceptible to resistance and transmissible variant emergence (Clark et al., 2021; Destras et al., 2022; Quaranta et al., 2022; Sabin et al., 2022). The emergence of resistance mutation thus impacting treatment efficacy is more likely if a patient has been exposed to specific antiviral drugs. In addition, it remains unclear if the small percent rebound occurrence (2%) observed with nirmatrelvir/r in the EPIC-HR (Evaluation of Protease Inhibition for COVID-19 in High-Risk Patients) trial, performed in the delta variant era, is underestimating a risk (Boucau et al., 2022; Rubin, 2022) that would be particularly of concern in patients harboring an impaired immune system and in the omicron era. In one recent case series, one out of 7 patients who had a virologic rebound also had an immunosuppressing condition (Boucau et al., 2022). Another recent case series (Coulson et al., 2022) revealed that all three patients with viral rebound were highly immunocompromised. This potentially raises concerns about the need of longer antiviral courses, especially in these patients.

Preclinical data have clearly demonstrated that virological efficacy is higher for combinations of existing antiviral drugs than single agents (Abdelnabi et al., 2021; Jeong et al., 2022; Li et al., 2022). To achieve the goal of changing the treatment guidelines in SARS-CoV-2-infected immunocompromised individuals, independent and academic clinical trials for drug combinations should be considered as an urgent, unmet research priority. Today, collaboration with industry to allow early access to antiviral drugs to be combined has been an objective still to be achieved (Bloomberg (Europe Edition), 2022). Certain potent monoclonal antibodies, such as bebtelovimab, cannot even be accessed for research or for routine care outside of the USA (Hentzien et al., 2022).

## Expert opinion

Treatment optimization has been truly transformational for other viral diseases [e.g., HIV/hepatitis C virus (Cohen et al., 2011)] and was only achieved when antiviral drug combinations became the mainstay. With few drugs currently available, the opportunity must be seized prior to the emergence of resistance to drugs deployed widely as monotherapies. Combinations of polymerase inhibitors and polymerase/protease inhibitors have proven highly successful for other viruses and in animal models for SARS-CoV-2 (Abdelnabi et al., 2021; Jeong et al., 2022). Thus, as drugs that are appropriate to combine are available, there is no good reason not to study them clinically. In addition to the opportunities that combinations present for a more potent antiviral response (individual benefit), there can be no doubt that the rate at which resistance emerges will also be reduced (public health benefit). Higher potency will result in a lower variability in sequences through a lower degree of replication. In addition, the probability of the occurrence of multiple mutations to drive resistance to multiple antivirals simultaneously is much lower than for a single agent (United States Food Drug Administration, 2021). This is particularly the case where concentrations achieved are close to the therapeutic efficacy threshold or in the case of low compliance.

It is incumbent upon the international research community and the pharmaceutical industry to pool knowledge and provide the critical information that the World Health Organization and country-level authorities so urgently require, as well as early diagnosis and increased access to vaccines and antiviral therapy. The resistance risk for existing drugs has been woefully understudied throughout development, making it extremely challenging to rationalize during policy development. Looking beyond efficacy, drug combinations will unquestionably reduce the rate at which resistance and new variants impacting treatment options emerge and could be made available and accessible to those in need if timely efforts are made.

In conclusion, we call for combination therapies to be tested in adequately powered clinical trials in the target population of immunocompromised patients, both in wealthy and in low-income countries where HIV-driven immunosuppression is prevalent. If higher efficacy is confirmed, the diversity of possible combinations will enable the tailoring of therapeutic options to individual patient needs (e.g., avoiding drug-drug interactions in transplant patients) as well as their specific regional context (e.g., oral-only combinations).

## Author contributions

MH and AC wrote the first manuscript draft. All authors critically reviewed the manuscript, validated the final version, and agreed to be accountable for the content of the work.

## Conflict of interest

The authors declare that the research was conducted in the absence of any commercial or financial relationships that could be construed as a potential conflict of interest.

## Publisher's note

All claims expressed in this article are solely those of the authors and do not necessarily represent those of their affiliated organizations, or those of the publisher, the editors and the reviewers. Any product that may be evaluated in this article, or claim that may be made by its manufacturer, is not guaranteed or endorsed by the publisher.
